# Phenytoin: A promising non-antibiotic drug for the topical treatment of digital dermatitis in dairy cows

**DOI:** 10.14202/vetworld.2021.2907-2912

**Published:** 2021-11-12

**Authors:** El-Sayed El-Shafaey, Mohamed A. Hamed, Eman Abo Elfadl, Naglaa A. Gomaa, Mohamed Abdo Rizk

**Affiliations:** 1 Department of Surgery, Anesthesiology, and Radiology, Faculty of Veterinary Medicine, Mansoura University, Mansoura 35516, Egypt; 2 Department of Veterinary Medicine, College of Agriculture and Veterinary Medicine, Qassim University, Buraydah 51452, Qassim, Saudi Arabia; 3 Department of Surgery, Anesthesiology, and Radiology, Faculty of Veterinary Medicine, Aswan University, Aswan, Egypt; 4 Department of Animal Husbandry and Development of Animal Wealth, Faculty of Veterinary Medicine, Mansoura University, Mansoura 35516, Egypt; 5 Department of Animal Medicine, Faculty of Veterinary Medicine, Kafrelsheikh University, Kafrelsheikh, Egypt; 6 Department of Internal Medicine and Infectious Diseases, Faculty of Veterinary Medicine, Mansoura University, Mansoura 35516, Egypt

**Keywords:** chlortetracycline, cows, digital dermatitis, phenytoin

## Abstract

**Background and Aim:**

Digital dermatitis (DD) is one of the most common causes of lameness in dairy cattle. It is seen in nearly all dairy herds across the world and has substantial welfare and economic implications. In this study, we aimed to investigate the efficacy of phenytoin sodium topical treatment on painful ulcerative stage of bovine digital dermatitis (BDD).

**Materials and Methods:**

In total, 45 Holstein-Friesian dairy cows with DD were randomly assigned to one of the three topical treatment trials (15 each): Saline solution (first treatment, negative control), chlortetracycline spray (second treatment, positive control), or phenytoin sodium powder (third treatment, positive control) (third treatment). On day 0 (pre-treatment) and on days 7, 14, 21, and 28 post-treatment, the response of DD-affected cows to the medications used was evaluated by measuring lesion depth and size, as well as the total clinical score (lameness, pain, and discomfort).

**Results:**

The cure rate in cows treated with phenytoin (86.66%) on day 28 was significantly improved compared to cows treated with either chlortetracycline (60%) or normal saline (6.66 %).

**Conclusion:**

Our findings highlight the superiority of phenytoin over the commonly used antibacterial agent, chlortetracycline, in the topical treatment of BDD, and subsequently suggest that phenytoin should be considered a suitable alternative treatment option for the treatment of BDD.

## Introduction

Digital dermatitis (DD) is a contagious infection that often affects cattle’s feet. It has been considered as one of the most common causes of lameness in dairy herds [1-3]. This infection has a great economic impact, resulting in a significant decrease in milk production, reproductive usefulness, and productive lifecycle of the affected cows [4]. The presence of confined lesions on the claw skin, particularly between the heels of the hind claws, is a symptom of DD [5]. Several factors are incriminated in the occurrence of DD in dairy cows, including *Treponema* bacteria species [6], environmental factors [5], farm management [7], and individual animal factors [3].

DD has been treated with a variety of chemical compounds in systemic, independent topical, and mass topical forms that involve a footbath [1,8,9]. Antibiotics have already shown their effectiveness against DD [10]; however, all currently used antibiotics are expensive, and drug residues have been detected in the meat of treated animals [11]. Subsequently, searching for more safe and cost-efficient drugs to treat DD in dairy cows is urgently required. In this regard, phenytoin (diphenylhydantoin) is a commercially available local dressing agent used for improving wound and ulcer healing in humans and various animal species [12-14].

The low cost and easy-to-use application of this drug together with its antibacterial effect on the connective tissue encourage us to evaluate for the 1^st^ time the efficacy of phenytoin in treating DD in dairy cows as a means of alternative therapy for the commonly used antibacterial agent that is, chlortetracycline spray.

## Materials and Methods

### Ethical approval and Informed consent

The Committee of Animal Welfare and Ethics of Mansoura University’s Faculty of Veterinary Medicine approved this investigation. According to the Egyptian Medical Research Ethics Committee (no. 14–126), all institutional and national guidelines for the care and use of animals were followed. The owner’s informed written consent was acquired.

### Study period and location

The study was conducted from October 2018 to June 2020 on dairy Holstein-Friesian cows with DD engaged in smallholder farms in Egypt’s El-Dakahlia province.

### Cows and housing

From 126 dairy Holstein-Friesian cows with DD engaged in a randomized clinical trial in smallholder farms (4-12 animals/farm) in Egypt’s El-Dakahlia province, 45 dairy Holstein-Friesian cows were randomly selected. The animals chosen were between 2 and 5 years old and weighed 450-550 kg. All cows were kept in free-stall systems with mattress padded cubicles and fed total mixed feeds. Cows were milked twice a day, and they were artificially inseminated. The average cow milk yield per year in the farms surveyed was 7600 kg energy-corrected milk. A skilled hoof trimmer used a transportable hydraulic trimming chute machine to perform claw trimming twice a year. However, footbaths at dairy farms were not included in this analysis.

### Study design

Our current investigation employed 45 Holstein-Friesian cows with active DD lesions (M2) in their hind claws over the bulb of the heel. Visual evaluation of the injured feet in the selected cows confirmed the presence of M2 lesions. To make this inspection easier, the rear feet were cleansed with a water hose and a nozzle, and the existing manure was removed using paper towels. The diameter, nature, and position of lesions, as well as the degree of pain in response to firm pressure and past treatment history, were all noted for the affected claw, which provided us with details regarding the respective lesions. The selected cows were randomly assigned to three treatment groups (15 each), as follows: The first group was treated with normal saline 0.9% (Otsuka Pharmaceutical Co., S.A.E., Egypt) and served as the negative control. The second group received chlortetracycline spray (CTC Spray GRVet, Netherland) and was used as the positive control. Finally, phenytoin sodium spray (Healosol 2%, Egyptian Company for Advanced Pharmaceuticals, Egypt) was administrated to the cows belonging to the third group. The treatment regimen and the follow-up healing process of the affected claws were performed using the following procedure: First, mechanical debridement and cleansing were performed to the affected claws with DD before the initiation of the topical treatment. The infected claws had necrotic tissues and granulomas, and any remaining damaged tissues were removed. The topical treatment was administered immediately after the claws were debrided, for a period of 72 h with sterile gauze and hoof tape. Consequently, bandages were exchanged during follow-up evaluations on days 7, 14, 21, and 28. The treatment was repeated an additional time during the 1^st^ week of the study. Then, the therapy was given once a week until the end of this research study on day 28. All cows were kept in a dry environment for 30 min after topical treatment before returning to the cubicle [15]. We considered that the hooves were healed when a full epithelial layer was formed on the lesion (M0) site with concurrent absence of pain during palpation. In contrast, the presence of pain or of an active lesion on day 28 post-treatment was considered to represent insufficient treatment. The healing progress of DD was documented weekly using a standard scoring system and a digital camera until the end of this study on day 28.

### Foot examinations

Visual and digital palpations were both used to evaluate DD lesions. Clinical examination was used to determine the stage, depth, and size of the lesion (Table-1) [2,10]. All clinical indicators of DD were measured and quantified by a single person who was blinded to the treatment process. The lesion stage was assessed using a scoring system that involved five stages (M0-M4) that have been previously described by Döpfer *et al*. [16]. A stainless steel investigator probe and a measuring tape (at the broadest area of the lesion) were used to determine the depth and size of the lesion, respectively. We considered that changes from any score to “no lesion” identified during the next observation represented resolution of DD lesions.

**Table-1:** Foot examination scores for subjective assessment of lesion parameters in Holstein-Friesian cows with digital dermatitis.

Parameters	Score and synopsis
Stage	0=The negative stage of disease comprises feet with normal digital skin (M0) 1=A lesion in its early stages (0-2 cm in diameter) that is not painful when palpated (M1) 2=The characteristic ulcerative stage, with a diameter of >2 cm and a palpable pain (M2) 3=After local therapy, when the lesion is covered by a scab, the healing stage begins (M3) 4=The chronic stage, which is characterized by surface growth that is not unpleasant to the touch (M4)
Depth	0 indicates that there is no lesion; 1 indicates that it is shallow; 2 indicates that it is proliferative; and 3 indicates that it is deep
Size	0 indicates that there is no lesion; 1 indicates that it is small (less than 1 cm); 2 indicates that it is medium (1-2 cm); and 3 indicates that it is large (more than 2 cm)

### Scores for clinical findings

One individual performed a subjective examination of the affected cows while walking and resting as a clinical finding score before and during treatment (Table-2). Lameness was graded on a scale from 0 to 3, with 0 indicating no lameness and 3 indicating significant lameness [17]. Foot retraction (mild, score 1), abnormal foot trembling (moderate, score 2), and/or non-weight-bearing were used to assess pain using rigid digital pressure with one thumb (severe, score 3) [18]. The discomfort was accurately tracked throughout the day by detecting variations in usual behavior, how it affected cow’s hunger, and changes in normal mood and lying down [19].

**Table-2:** The clinical finding scores for subjective assessment of clinical parameters in Holstein-Friesian cows with digital dermatitis.

Parameters	Score and description
Lameness	0 indicates that everything is as it should be. Cow stands with a level back and walks. The gait appears to be normal 1=Cow is mildly lame, standing level backed but walking with an arched back. Gait is normal 2=Moderately lame, with an arched back when standing or walking. Short strided gait 3=Lame, arch back visible at all times, and one deliberate step at a time stride. One or more legs/feet are preferred by the cow 4=Severely lame, Cow shows an inability or strong aversion to bearing weight on one or more limbs/feet
Pain	0 indicates no symptoms, 1 indicates mild symptoms, 2 indicates moderate symptoms, and 3 indicates severe symptoms
Discomfort	0 indicates comfort; 1 indicates discomfort

### Statistical analysis

GraphPad Prism, a statistical software application, was used to conduct the statistical analysis of this study (GraphPad Prism for Windows version 5.0, GraphPad software Inc., USA). The Kruskal–Wallis test was used to compare two groups at different points in time (days 0, 7, 14, 21, and 28), whereas the Mann–Whitney non-parametric t-test was performed to analyze the differences between the two groups. At p<0.05, differences between the median and range were considered significant. The Chi-square test was performed to examine the curative impact (percent) of topical chlortetracycline and phenytoin spray treatments. The results were considered significant when p<0.05 was considered.

## Results

**Figure-1:**
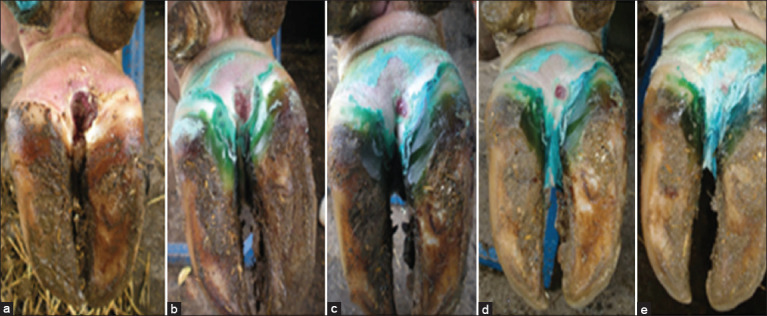
Pre- and post-phenytoin therapy clinical findings of a digital dermatitis (DD) lesion on a Holstein-Friesian cow’s hind claw. On day 0, (a) a large (>2 cm) active DD (M2) lesion. (b) The lesion’s dryness (day 7). (c) Has a dark scab that is mending (day 14). (d) Significant reduction in the depth and extent of the lesion (day 21). (e) Total resolution of DD lesions (day 28).

To assess the response of the DD-affected cows to the drugs used, this study determined the lesion depth and size, clinical sum score, and curative percentages. Pertaining to the lesion depth and size, topical application of phenytoin induced a significant decrease (p<0.05) in both values followed by the chlortetracycline-treated cows, especially on days 14, 21, and 28 (p<0.05) post-treatment compared with normal saline-treated cows (Table-3 and Figure-1). In addition, a significant decrease (p<0.05) in lameness and pain scores was observed in cows treated with topical phenytoin compared with cows treated with chlortetracycline and normal saline, especially on days 14, 21, and 28 (p<0.05) post-treatment (Table-4). Furthermore, treatment of cows with phenytoin and chlortetracycline resulted in a significant decrease (p<0.05) in the discomfort score, especially on days 21 and 28 post-treatment, compared with normal saline-treated cows (Table-4). Interestingly, cows treated with phenytoin exhibited better curative efficacy (86.66%) on day 28 in comparison with cows treated with chlortetracycline (60%) and normal saline (6.66%), respectively (Table-5).

**Table-3:** The effect of topical chlortetracycline and phenytoin spray on the depth and size of lesions (median and range).

Treatment	Day 0	Day 7	Day 14	Day 21	Day 28
Lesion depth					
T^1^	2 (1-3)	2 (1-3)	2 (1-3)*	2 (1-3)*	2 (1-3)*
T^2^	2 (1-3)	2 (1-3)	2 (1-3)*	1 (0-3)	1 (0-2)
T^3^	2 (1-3)	1 (1-3)	1 (0-3)	0 (0-2)	0 (0-1)
p-value	0.741	0.189	0.345	**<0.0001**	**<0.0001**
Lesion size					
T^1^	2 (0-3)	2 (1-3)*	2 (0-3)*	2 (1-3)*	1 (0-3)*
T^2^	2 (1-3)	2 (0-2)*	1 (1-2)	1 (0-2)	1 (0-1)*
T^3^	2 (1-3)	1 (1-2)	1 (0-3)	0 (0-2)	0 (0-1)
p-value	0.544	**0.040**	**0.001**	**0.002**	**0.001**

* Significant differences between median of lesion depth. T1=Animals treated with normal saline; T2=Animals treated with chlortetracycline; T3=Animals treated with phenytoin, Bold values indicate the values with significant differences in comparison with the control group

**Table-4:** Lameness, pain, and discomfort scores after topical therapy with chlortetracycline and phenytoin spray (median and range).

Treatment	Day 0	Day 7	Day 14	Day 21	Day 28
Lameness score					
T^1^	3 (1-4)	2 (1-3)	2 (0-2)*	2 (0-2)*	1 (0-2)*
T^2^	2 (2-4)	2 (1-3)	1 (1-3)*	1 (0-2)	1 (0-1)
T^3^	1 (1-1)	0 (0-1)	0 (0-1)	0 (0-1)	0 (0-1)
p-value	0.533	0.562	**<0.0001**	**<0.0001**	**<0.0001**
Pain score					
T^1^	2 (1-3)	2 (1-3)*	2 (0-3)*	2 (1-3)*	1 (0-3)*
T^2^	2 (1-3)	2 (0-2)*	1 (1-2)	1 (1-1)*	1 (0-1)*
T^3^	2 (1-3)	1 (1-2)	1 (1-1)	0 (0-1)	0 (0-1)
p-value	0.844	**0.040**	**<0.0001**	**<0.0001**	**0.001**
Discomfort score					
T^1^	1 (1-1)	1 (1-1)*	1 (0-1)*	1 (0-1)*	1 (0-1)*
T^2^	1 (1-1)	1 (1-1)*	1 (0-1)*	0 (0-1)	0 (0-1)
T^3^	1 (1-1)	0 (0-1)	0 (0-1)	0 (0-1)	0 (0-1)
p-value	1	**<0.0001**	**0.001**	**0.001**	**0.004**

* Significant differences between median of lameness. T^1^=Animals group treated with normal saline; T^2^=Animals group treated with chlortetracycline; T^3^=Animals group treated with phenytoin, Bold values indicate the values with significant differences in comparison with the control group

**Table-5:** Topical therapy with chlortetracycline and phenytoin spray has a curative outcome (%).

Treatments	Day 0	Day 7	Day 14	Day 21	Day 28	Chi-square	p-value
T^1^	0	0	0	0	1 (6.66)	4.05	0.398^n.s^
T^2^	0	0	2 (13.33)	5 (33.33)	9 (60)	23.37	0.0001**
T^3^	0	1 (6.66)	5 (33.33)	10 (66.66)	13 (86.66)	35.64	<0.0001**
Chi-square	1	2.045	6.428	15	19.92	Total chi for all=2.04	
p-value	0	0.359^n.s^	0.040*	0.00060**	<0.0001**	p=0.915	

* Significant differences between percent of curative effect. T^1^=Animals group treated with normal saline, T^2^=Animals group treated with chlortetracycline, T^3^=Animals group treated with phenytoin. n.s.=Non-significant

## Discussion

Individual antibiotic topical treatment is the most well-documented method for the treatment of bovine DD (BDD) [17]. However, this method has been associated with the development of bacterial resistance with concurrent hazards related to environmental contamination [11]. Subsequently, alternative therapeutic processes have become an urgent demand. In this regard, there is a paucity of the effectiveness of non-antibiotic preparations for the treatment of BDD. As a result, this study clinically assessed the impact of phenytoin, a non-antibiotic medicine, on BDD treatment for the 1^st^ time. In particular, our findings revealed that phenytoin treatment was more effective compared to chlortetracycline treatment on day 28 in terms of cure rate, lesion size reduction, and pain relief.

In this study, phenytoin displayed better and more reliable outcomes for BDD care within 14 days of treatment, as shown by the substantially improved scores of lesion depth, size, and clinical findings. These results are consistent with the previous studies on both humans and rodents [12-14]. The influence of phenytoin on treating DD might be attributed to an increase in fibroblast proliferation, which, in turn, hinders the action of collagenase, encourages collagen deposition, improves the formation of granulation tissue, reduces bacterial contamination, and thus limits the formation of wound exudates [12,13]. Importantly, the low cost and ease of use of phenytoin make it a good candidate for clinical use[12-14].

The success of the topical therapy depends on the anatomical location and stage of DD lesions in dairy cows [18,19]. Each DD lesion in our investigation was active and widespread in the hind claws, with a typical location on the planter skin above the heel bulb. This could be due to the development of intimate communication between heel bulbs, which are more likely to constantly stay moist, thus favoring the generation of DD, particularly when it comes to M2 lesions [5]. Typically, the lesion site above the heel bulb keeps the drug in direct contact with the active lesion for a period of 3 weeks, allowing the lesion to dry out and recover. Active DD cows exhibited varying degrees of lameness, ranging from mild to severe [3,20]. Similar effects were observed on day 0 in the current investigation, which subsequently decreased over the course of the treatment, especially in cows given phenytoin.

The severity of the lesion, irritability, and high sensitivity of the damaged region contribute to the pain and discomfort associated with DD-affected cows[4]. All affected cows in this current study exhibited increased discomfort scores on day 0, scores that were gradually and significantly decreased following the prescribed therapy, especially with phenytoin. The M2 lesions are confined regions of ulcerative, erosive dermatitis, which could explain this observation. Because of the dermis involvement, lesions tend to become unpleasant, and the afflicted animals become lame and uncomfortable. When comparing phenytoin-treated cows with cows treated with chlortetracycline antibiotics on day 14 after treatment, discomfort was found to be dramatically reduced. The capacity of phenytoin to suppress the inflammatory response and its effect on membrane stabilization [20] may explain the soothing effect on these cows.

Hydraulic debridement and trimming of the claw of the diseased leg before the application of topical medicine were found to enhance DD healing by eliminating all visible filth and exudation from the affected skin region, exposing the lesions to air, and thus allowing them to dry out. These findings corroborate previous findings [2,21]. Furthermore, keeping the cows on a dry surface with a light hoof bandage applied to cover the lesion improves the healing rate by allowing the damaged tissue to be exposed to the topical therapy for a longer period of time. The results were in line with what was expected [22].

Interestingly, phenytoin exhibited the best healing effect on the lesion scores on day 28 in comparison with the chlortetracycline solution. Such findings might be attributed to the possible bacterial resistance to chlortetracycline, but also to the stimulating effect of phenytoin on both lymphocytic chemotaxis and angiogenesis [13,23].

Importantly, in this current study, phenytoin exhibited a high and rapid curative effect (86.66%) on day 28 after treatment. Compared to the results recorded for topical antibiotics in a previous study, in which cure rates ranged between 60 and 70% on day 30 post-treatment, our study reports improved and more promising findings [19,24,25]. As a result, phenytoin appears to be a viable alternative to antibiotic therapy of M2 BDD lesions.

In this present study, precautions were performed, which have substantially aided the healing process, including hydraulic debridement and trimming of the claw of the affected leg before topical drug application, and removing all visible dirt and exudate from the affected skin region, exposing the lesions to air for drying. Furthermore, we kept the affected cows on a dry surface with the use of a light hoof bandage on the affected claw, a process that improves the healing rate by exposing the affected tissue to the topical medication for a longer period of time. Subsequently, we expect that the topical application of phenytoin on the affected claw with DD together with the implementation of these precautionary measures can act as a promising approach for the treatment of cows with DD.

## Conclusion

To the best of our knowledge, there have been no other published studies examining the influence of phenytoin, a non-antibiotic medicine, on BDD treatment. Phenytoin demonstrated substantial improvement in lesion depth and size, lameness, pain, and discomfort scores compared with cows treated with chlortetracycline, which is a commonly used antibiotic. In addition, cows treated with phenytoin exhibited better curative efficacy on day 28 compared with cows treated with chlortetracycline. Our results show that phenytoin has a potential therapeutic efficacy when administered topically to dairy cows with DD, suggesting that this medicine could replace antibiotics in the treatment of dairy cows with DD.

## Authors’ Contributions

EE, MAH, and MAR: Conceived and designed the experiments. EE, MAH, NAG, and MAR: Performed the experiments. MAH and EAE: Analyzed the data. EE, MAH, NAG, and MAR: Contributed reagents/materials/analysis tools. EAE and MAH,: Drafted the manuscript. All authors read and approved the final manuscript.
